# Effects of Physical and Social Activity on Physical Health and Social Inclusion of Elderly People

**Published:** 2019-10

**Authors:** Stevo POPOVIC, Bojan MASANOVIC

**Affiliations:** Faculty for Sport and Physical Education, University of Montenegro, Niksic, Montenegro

## Dear Editor-in-Chief

The concept of social inclusion describes the taking of people's participation in common social activities, and the opposite is the concept of social exclusion, situations in which community members are separated from most of those who normally participate in a range of social activities, including employment, education and leisure (1).

The fact is that 11% of adults are not able to exercise friendship experience, to perform useful activities in society, and to hope for a better future (2), while the problem is much more pronounced as old age arrives. If organized physical activity can reduce social exclusion in the elderly, and numerous previous studies confirm the positive impact of physical activity on cognitive abilities, social and physical development and health (3), the meaning of this study is very reasonable, and was reflected in the impact assessment of organized physical activity on the social inclusion of older people.

A sample of 60 subjects covered by this study is divided into three groups (two experimental and one control). For the first experimental group (15 respondents), a physical exercise program lasting 6 months was organized; for the second experimental group (15 respondents), a program that lasted for 6 months was also organized, in which the respondents had the opportunity to socialize in all segments of life, except in joint physical activities; third group, control group (30 respondents) performed normal activities.

The criterion for inclusion in the experiment was that respondents do not have health problems, that they do not engage in sports activities for a longer period of time, and that they are older than 55 and younger than 70 years. The experimental exercise program was adapted to the age and abilities of the respondents. The survey, which was conducted, was aimed at determining the level of social inclusion at the beginning and end of the experimental program, and the questionnaire "Social Inclusion Scale (SIS)" was applied, in which social isolation, social relations and social acceptance were examined separately (4). To test the difference in the degree of social involvement between the groups before and after the end of the experimental treatment, MANOVA was employed.

The results of this study show that the applied experimental programs have influenced the social inclusion of older people in different ways, depending on the group and group, as well as from category to category. Namely, in the case of social isolation and social acceptance, there are no differences between groups, so it cannot be concluded that organized physical exercise influenced when these two categories were concerned. However, on the other hand, in social relations, statistically significant differences were found in favor of the experimental group that attended the program of organized physical exercise in relation to the other two groups, primarily the control group. Therefore, the program of organized physical activity for the elderly has proved to be useful for improving social relations in older people. Although previous studies have led to the conclusion that organized physical activity has a positive impact on social inclusion (5), this study confirms, or recognizes, certain subcategories of social inclusion that have influence, as well as those that do not. It is interesting to point out that previous research emphasized the fact that every organized social activity positively influences the suppression of loneliness and on the social inclusion of the individual (6), nevertheless this study does not determine any positive effects in the group that had exclusively organized joint activities which did not contain elements of physical activity, and thus opened up a series of new research questions. Before turning to the limitations of this study, it is worth mentioning that the study he conducted (7) found that organized physical exercise did not have an impact on the social involvement of young people, which opens up a number of research questions when different age categories are concerned.

The possible limitations of this study are that the sample of the respondents was small and that most of the respondents were interested in taking part in an experimental group with physical activity. Also, the limitation can be seen in the fact that the experimental program lasted only six months, that is, it takes more time to influence social inclusion, and there remains an open question when comparing a study with younger and elderly people, is the impact which is determined by the social relationships of older people caused by the age or duration of the experimental program.
